# Controlled Quenching of Agarose Defines Hydrogels with Tunable Structural, Bulk Mechanical, Surface Nanomechanical, and Cell Response in 2D Cultures

**DOI:** 10.1002/adhm.202300973

**Published:** 2023-07-09

**Authors:** Francesco Piazza, Pietro Parisse, Julia Passerino, Eleonora Marsich, Luca Bersanini, Davide Porrelli, Gabriele Baj, Ivan Donati, Pasquale Sacco

**Affiliations:** ^1^ Department of Life Sciences University of Trieste Via Licio Giorgieri 5 Trieste I‐34127 Italy; ^2^ NanoInnovation Lab Elettra‐Sincrotrone Trieste S.C.p.A. Trieste I‐34149 Italy; ^3^ Istituto Officina dei Materiali (IOM‐CNR) Area Science Park Trieste I‐34149 Italy; ^4^ Department of Medicine Surgery and Health Sciences University of Trieste Piazza dell'Ospitale 1 Trieste I‐34129 Italy; ^5^ Optics11 Life Hettenheuvelweg 37–39 Amsterdam 1101 BM The Netherlands; ^6^ Interdepartmental Centre for Advanced Microscopy Department of Life Sciences University of Trieste Via Alexander Fleming 31/A Trieste I‐34127 Italy

**Keywords:** agarose, cell activity, controlled cooling, hydrogel networks, surface/bulk mechanical properties

## Abstract

The scaffolding of agarose hydrogel networks depends critically on the rate of cooling (quenching) after heating. Efforts are made to understand the kinetics and evolution of biopolymer self‐assembly upon cooling, but information is lacking on whether quenching might affect the final hydrogel structure and performance. Here, a material strategy for the fine modulation of quenching that involves temperature‐curing steps of agarose is reported. Combining microscopy techniques, standard and advanced macro/nanomechanical tools, it is revealed that agarose accumulates on the surface when the curing temperature is set at 121 °C. The inhomogeneity can be mostly recovered when it is reduced to 42 °C. This has a drastic effect on the stiffness of the surface, but not on the viscoelasticity, roughness, and wettability. When hydrogels are strained at small/large deformations, the curing temperature has no effect on the viscoelastic response of the hydrogel bulk but does play a role in the onset of the non‐linear region. Cells cultured on these hydrogels exhibit surface stiffness‐sensing that affects cell adhesion, spreading, F‐actin fiber tension, and assembly of vinculin‐rich focal adhesions. Collectively, the results indicate that the temperature curing of agarose is an efficient strategy to produce networks with tunable mechanics and is suitable for mechanobiology studies.

## Graphical abstract

Controlled quenching (cooling) of agarose after heating by temperature‐curing steps is an efficient material strategy for producing hydrogel networks with tuneable structural/mechanical properties. Fast cooling promotes the accumulation of agarose on the surface and determines the local stiffness; slow cooling provides a more homogeneous distribution of agarose with less stiffness. This clearly affects the mechanosensing of hydrogels by cells.

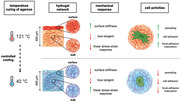

## 1 Introduction

Agarose is a linear heteropolysaccharide extracted from marine red algae. It consists of repetitions of the disaccharide agarobiose, with alternation of d‐galactose and 3,6‐anhydro‐l‐galactopyranose units linked by *α*‐(1→3) and *β*‐(1→4) glycosidic bonds.^[^ ^1^ ^]^ Agarose can form thermoreversible physical hydrogels by hydrogen bonding. At high temperatures and in aqueous solutions, agarose dissolves and forms random coil structures. When cooled, it begins to form helical motifs and bundles that allow gelation.^[^ ^2^ ^]^ The gelation process depends, therefore, on temperature, but also the molecular weight and concentration of the polymer.^[^ ^3^, ^4^ ^]^ At very low agarose concentrations, the polymer chains aggregate in a helix conformation and form clusters. As the polymer concentration increases, a sol–gel transition occurs in which the clusters combine via fibrillar bundles of different compositions and form a hydrogel.^[^ ^5^, ^6^ ^]^ The overall process is thought to occur in three phases: induction, gelation, and pseudo‐equilibrium phase.^[^ ^7^ ^]^ Upon formation, agarose hydrogels have shown different mesh sizes depending on the biopolymer concentration, ranging from about 500 to 100 nm.^[^ ^8^ ^,^ ^9^ ^]^

Given the biocompatibility of agarose, and more broadly, agarose hydrogels, they can be used as a platform for tissue engineering and mechanobiology studies.^[^ ^1^, ^10^, ^11^, ^12^, ^13^, ^14^, ^15^, ^16^, ^17^, ^18^ ^]^ Understanding the structural and mechanical properties of these systems is therefore crucial to the intended need. It has been shown that the failure stress, shear modulus, and elastic modulus scale with the molecular weight of agarose.^[^ ^3^ ^]^ Conversely, the failure stress decreases as the average molecular weight decreases, suggesting that the network structure is connected at shorter intervals when the chain length is shorter.

Recently, it has been shown that agarose hydrogels can exhibit a stiffening or softening response under oscillatory mechanical stimulation, depending on the experimental sample preparation and rheological measurement setup.^[^ ^19^, ^20^ ^]^ While the stiffening is attributed to the semiflexible nature of the agarose fibrils and their geometric arrangement, which should be below the central‐force isostatic critical connectivity, less information is available for the softening behavior that may occur due to the progressive unwinding of bundles.^[^ ^10^ ^]^ Nevertheless, the residual methylation pattern has been shown to play a key role in modulating the onset of softening, which has fundamental implications for the mechanical sensing of the agarose substrates by the cells.^[^ ^21^ ^]^

Standard protocols for the preparation of agarose hydrogels involve dissolving the agarose at high temperature and then rapidly cooling it to room temperature. However, this uncontrolled quenching could have an important influence on the final hydrogel structure, given the known particular temperature‐assisted gelation of the biopolymer. The effects of temperature curing of agarose solutions on the structure, mechanics, and biological properties of the final biomaterials have generally been overlooked. Here, we describe the effects of temperature curing of agarose in relation to the final hydrogel structure, and mechanical and biological response. By combining microscopy, standard mechanical analyses, and advanced nanomechanical tools, we are able to demonstrate that temperature curing of agarose plays a crucial role in modulating the final hydrogel properties and the response of cells in 2D culture.

## 2 Results

### 2.1 Temperature Curing of Agarose Defines Hydrogels with Different Physical Structures

**Figure 1:**
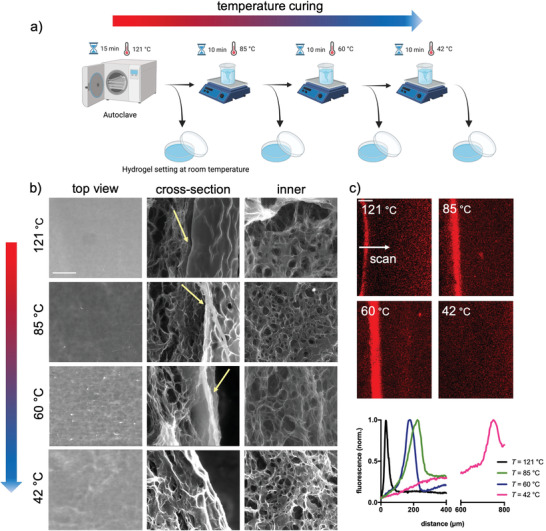
Temperature curing of agarose solution defines hydrogels that have different networks. a) Cartoon recapitulating the experimental setup used in this study, which involves controlled cooling steps before the hydrogel forms at room temperature. After gelation, agarose hydrogels are incubated for an additional 24 h at 37 °C to equilibrate the network.^[^ ^7^ ^]^ b) Scanning electron micrographs in the environmental state of agarose hydrogels obtained at different curing temperatures: top view (left), cross‐section (middle), and inner part of the hydrogel (right); the yellow arrows indicate the agarose abundance; the scale bar is 100 µm. c) Confocal scanning electron microscopy of hydrogels prepared at different curing temperatures. The hydrogels were prepared with Atto Rho101 NHS ester‐labeled agarose (20% w/w) and sectioned to visualize the internal structure. The scan starts from the surface boundary and proceeds toward the inner part of the hydrogel as indicated in the images; the scale bar is 100 µm. The agarose profile (normalized fluorescence intensity) is shown in the plot. Two hydrogels for each experimental condition were analyzed.

In the first part of this work, we investigated the effect of temperature curing of agarose on the structural properties of the corresponding hydrogel networks. We used a temperature‐controlled experimental setup to cure agarose before hydrogel formation. First, the agarose is dispersed in deionized Milli‐Q water and autoclaved at 121 °C for 15 min. Then it is cured at different temperatures as schematically shown in the cartoon in **Figure** **1**a. Apart from 121 °C, which is the case of agarose solutions quenched immediately at room temperature after autoclaving, a controlled cooling approach with steps of ≈20 °C was selected to investigate the effect of thermal curing. After the temperature cycle is completed, the hydrogels were analyzed by scanning electron microscopy under environmental conditions (Figure 1b). Looking at the hydrogels in the top view shows that the curing temperature plays a role. While a clear agarose film can be seen when cured at 121 °C, this progressively disappears as the curing temperature is lowered. This is confirmed by cross‐section analyses of the same hydrogel networks, which show the abundance of polymer on the surface of the hydrogels in the case of the 121 °C sample, which becomes fewer and fewer as the curing temperature decreases. It is noticeable that the inner part of the hydrogel networks has an almost porous structure in all samples examined as expected.^[^ ^8^, ^9^ ^]^ To strengthen our findings, we assembled the hydrogels with labeled agarose and observed the distribution of biopolymer throughout the networks using confocal microscopy (Figure 1c). Moving from the surface boundary to the inner part of the hydrogel, we observe a sharp fluorescence peak in the first 100 µm of depth, followed by an almost constant amount of agarose up to 400 µm in the case of curing at 121 °C, indicating a clear accumulation of biopolymer at the hydrogel surface. A broader inner peak is noticeable in hydrogels obtained by curing agarose at 85 and 60 °C. The fluorescence peak is lost when cured at 42 °C, suggesting that the agarose is more evenly distributed at this temperature. In the latter case, the amount of agarose is at a greater depth around 800 µm. The structural stability of the hydrogels was finally evaluated by the swelling properties over a period of three days and shows very little swelling and almost no influence of the curing temperature (Figure S1, Supporting Information). Overall, these results indicate that the different quenching causes inhomogeneities in the final networks that can be mostly recovered in the first 400 µm depth upon lowering the curing temperature of the agarose solution.

### 2.2 Temperature Curing of Agarose Defines Hydrogels with Different Bulk and Surface Mechanical Properties

**Figure 2:**
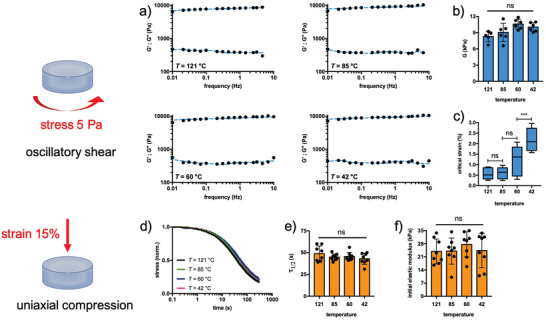
The bulk mechanical response of agarose hydrogels shows the same stiffness and viscoelasticity, but different linear stress–strain behavior. The agarose hydrogels obtained through a controlled quenching were subjected to oscillatory mechanical stimulation by rheometry or uniaxial compression. a) Sample‐case mechanical spectra of agarose hydrogels: the black dots represent the experimental points, while the solid blue line represents the fit by Equations (1) and (2) in the manuscript. b,c) Shear modulus and critical strain, γ_crit_, where the linear stress–strain response of the materials ends. Frequency sweep measurements were performed with a constant stress of 5 Pa, while the stress sweep analyses were performed with a constant frequency of 1 Hz. d–f) Normalized stress‐relaxation curves, the time needed to relax the stress to half, *τ*_1/2_, and initial elastic modulus of agarose hydrogels under uniaxial compression. The stress relaxation experiments were performed by applying a constant strain of 15% for 300 s, while the initial elastic modulus was calculated in the range of 1–3% of the linear stress–strain range. Data in panels (b,e,f) are reported as mean ± s.d., *n* = 5–8 hydrogels analyzed for each experimental condition; data in panel (c) are reported as box and whiskers (min to max), *n* = 5–6 hydrogels analyzed for each experimental condition. Statistics: ^***^, *p* < 0.001; ns, not significant (one‐way ANOVA followed by Tukey's Multiple Comparison post hoc test).

Next, we performed an in‐depth mechanical characterization of the hydrogels prepared with agarose solutions cured at different temperatures. First, we investigated the bulk mechanical response by standard rheometry and uniaxial compression. To obtain information on the viscoelastic properties, oscillating shear stress was applied first. Frequency sweep analyses reveal very similar mechanical spectra among the hydrogels studied, with the elastic modulus, *G*′, being significantly higher than the viscous modulus, *G″*, for three decades of the frequencies analyzed (**Figure** **2**a). Moreover, *G*′ is weakly frequency dependent, indicating that the hydrogels are strongly elastic rather than viscous. This is confirmed by calculating the loss tangent at 1 Hz, which is very low (≈4%). However, no effect of curing temperature is observed on the overall viscoelasticity of hydrogels (Figure S2, Supporting Information). We modeled the frequency sweep data using a generalized Maxwell model involving a combination of spring/dashpot elements plus a purely elastic spring,^[^ ^22^ ^]^ according to the following equations

(1) $$G^{\prime }=\;G_{e}+\;\sum_{i\;=\;1}^{n}G_{i}\;\frac{{\left(\lambda_{i}\;\omega \right)}^{2}}{1+{\left(\lambda_{i}\;\omega \right)}^{2}};\;\;G_{i}=\;\frac{\eta_{i}}{\lambda_{i}}\;$$

(2) $$G^{\prime \prime }=\;\sum_{i\;=\;1}^{n}G_{i}\;\frac{\lambda_{i}\;\omega }{1+\;{\left(\lambda_{i}\;\omega \right)}^{2}};\;\;G_{i}=\;\frac{\eta_{i}}{\lambda_{i}}\;$$

where *n* is the number of Maxwell elements considered, *G*_i_, *η*_i_, and *λ*_i_ represent the spring constant, the dashpot viscosity, and the relaxation time of the *i* ^th^ Maxwell element, respectively. *G*_e_ is the spring constant of the last Maxwell element which is supposed to be purely elastic. The use of the generalized Maxwell model to describe agarose‐based hydrogels enables us to calculate the shear modulus, *G*, as

(3) $$G\;=\;G_{e}+\;\sum_{i\;=\;1}^{n}G_{i}\;$$

The experimental data presented in Figure 2b show that the curing temperature of agarose has no effect on the overall stiffness of the resulting hydrogels, as the shear modulus is ≈9 kPa for all samples examined. We also performed long stress sweep experiments in which we gradually increased the applied stress using a constant frequency of 1 Hz. Again, hydrogels made from agaroses cured at different temperatures show a similar response with the typical softening behavior at large deformations (Figure S3, Supporting Information).^[^ ^10^, ^19^ ^]^ Experimental points from long stress sweep experiments are modeled, from the phenomenological point of view, using Equation (4)

(4) $$\sigma \;=\frac{G_{0}}{1+b\gamma }\;\gamma \;$$

where *G*_0_ corresponds to the shear modulus at *γ*_→ 0_ and *b* is a fitting parameter. The critical strain, *γ*_crit_, which marks the onset of the non‐linear behavior, is arbitrarily determined as Equation (5)

(5) $$\gamma_{\mathrm{crit}}:\frac{\sigma }{G_{0}\cdot \gamma }\;=\;0.95$$

Interestingly, the curing temperature affects the critical strain at which strain softening originates, exponentially increasing moving from 121 to 42 °C‐cured agarose (Figure 2c). This is confirmed when hydrogels are prepared using the phosphate‐buffered saline (PBS) buffer, relevant for biological applications, and a second agarose with different physicochemical properties (*T*_gel_ = 40 °C; rotational viscosity at 60 °C = 24 mPa s; total methylation = 12.8%; agaropectin content = 1.1% w/w; Figure S4, Supporting Information). Therefore, this typical behavior can be considered universal for agarose hydrogels. Uniaxial compression experiments were also run to provide additional pivotal information. Hydrogels obtained from agarose cured at different temperatures were strained up to 15% and the stress relaxation was monitored over time (Figure 2d).^[^ ^23^ ^]^ The stress relaxation curves show a very similar trend, with a clear relaxation of the stress over time confirming the viscoelastic nature of the hydrogels. By calculating the time needed to relax the stress to half, *τ*_1/2_, we have found that hydrogels obtained are fast‐relaxing, showing *τ*_1/2_ around 50 s (Figure 2e). Also in the calculation of the initial elastic modulus, we do not observe any differences between the analyzed samples (Figure 2f). Collectively, this body of evidence indicates that agarose solutions cured at different temperatures define hydrogels with the same bulk stiffness and viscoelasticity but different critical strains at which softening originates.

**Figure 3:**
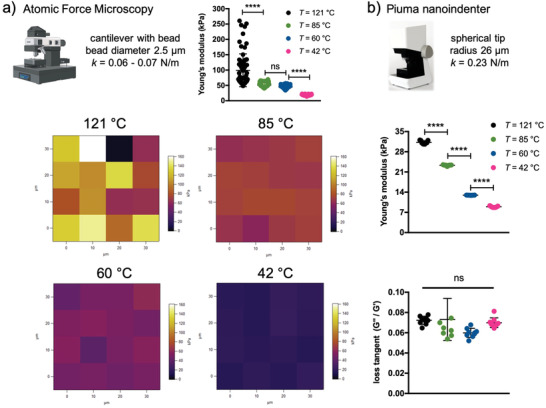
The surface mechanical response of agarose hydrogels shows different stiffness, but the same viscoelastic behavior. a) Atomic force microscopy analysis of surface hydrogels obtained from agarose cured at different temperatures. Dependence of Young's modulus as a function of curing temperature and corresponding force maps. b) The hydrogel surface was also analyzed with a Piuma nanoindenter, which has a different tip configuration. Dependence of Young's modulus and loss tangent recorded at a frequency of 1 Hz as a function of curing temperature. Data are given as mean ± s.d., *n* = 72–83 determinations for the analysis with the atomic force microscopy and *n* = 9 determinations for the analysis with the Piuma nanoindenter. Statistics: ^****^, *p* < 0.0001; ns, not significant (one‐way ANOVA followed by Tukey's Multiple Comparison post hoc test).

Next, we performed advanced nanomechanical tests on the hydrogel surface. For this, we used atomic force microscopy (AFM) equipped with a bead in the cantilever with a diameter of 2.5 µm and a Piuma nanoindenter using a tip with a radius of 26 µm. In **Figure** **3**a, we show the force maps obtained after evaluating Young's modulus of the different samples using the typical indentation curves (Figure S5, Supporting Information). The stiffness of the hydrogel surface decreases when the curing temperature of the agarose solutions is lowered. This becomes much clearer when hydrogels are analyzed with the Piuma Nanoindenter, which shows a progressive decrease of Young's modulus from ≈30 kPa in the case of the 121 °C sample to ≈8 kPa for 42 °C sample (Figure 3b). The discrepancy between the absolute values of Young's modulus when comparing the AFM results with the Piuma nanoindenter can safely be attributed to the different contact radii of the tip.^[^ ^24^ ^]^ Strikingly, the mechanical properties of the surface are similar in terms of viscoelasticity, as shown by the calculation of the loss tangent at 1 Hz through dynamic mechanical analyses. From all these results, we conclude that agarose solutions cured at different temperatures define hydrogels with different nanomechanical surface properties in terms of stiffness but not viscoelasticity.

### 2.3 Investigation of the Roughness and Wettability of the Surface of Hydrogels

**Figure 4:**
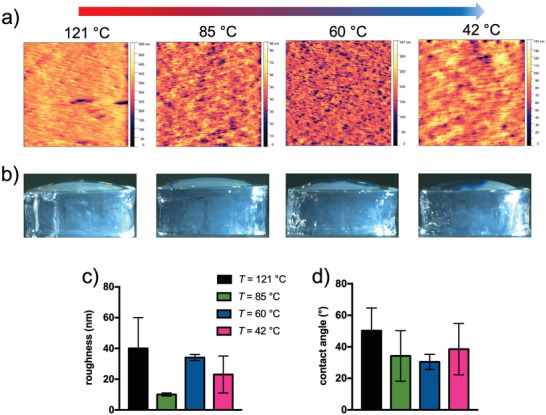
Hydrogels of agarose cured at different temperatures exhibit a porous and hydrophilic surface. a) 20 × 20 µm^2^ topographic images obtained through atomic force microscopy working in contact mode using NSC‐36 cantilevers (radius of curvature < 10 nm, spring constant 0.6 < *k* < 2 N m^−1^) of the hydrogels prepared with agarose solutions cured at different temperatures; the color scale on the right refers to the pore depth, which ranges from 0 to ≈560 nm depending on the sample analyzed. b) Representative images of water droplets atop hydrogels from agarose solutions cured at different temperatures. c) Roughness results upon image processing; data are reported as mean ± s.d., *n* = 4–5 images analyzed. d) Contact angle results upon image processing; data are reported as mean ± s.d., *n* = 6 droplets analyzed.

**Figure** **4**a shows the topographic images of the hydrogels prepared with agarose cured at different temperatures. The 20 × 20 µm^2^ images exhibit a morphology characterized by a porous structure with pores whose depth, shape, and lateral dimensions vary slightly among the different hydrogels analyzed. Due to the low homogeneity of the surface, the determination of the precise size of the pores is hampered. Instead, we analyzed the mean square roughness in different areas of the hydrogel samples (Figure 4c). Apart from the agarose hydrogel, whose solution was cured at 85 °C and has a more homogeneous surface with lower roughness, the other hydrogels are more bumpy, which is reflected in a higher roughness that mildly increases as a function of the curing temperature. Contact angle measurements were made to also investigate the wettability of the hydrogel surface (Figure 4b). When deionized water was applied to the hydrogels, the drops appeared flattened. The results of the contact angle after image processing exhibit clear hydrophilicity (contact angle < 90°) regardless of the type of hydrogel (Figure 4d).^[^ ^25^ ^]^ Overall, these results suggest that the curing temperature of agarose solutions defines hydrogels with hydrophilic porous surfaces and variable roughness.

### 2.4 The Curing Temperature of Agarose Impacts on Cell Adhesion, Spreading, and Focal Adhesion Assembly Atop Hydrogels

**Figure 5:**
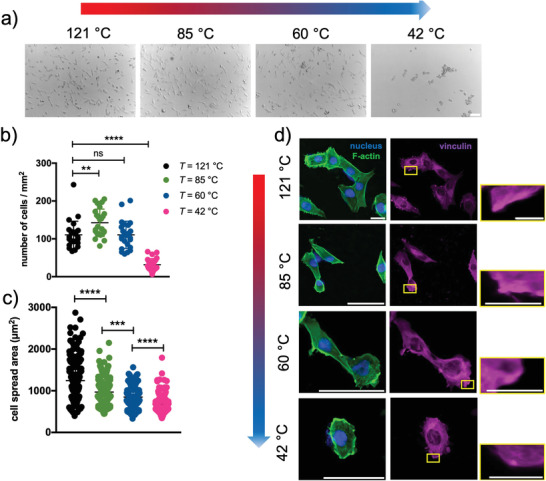
The adhesion, spreading of cells, and focal adhesion assembly are influenced atop hydrogels from different curing temperatures of agarose. a) Representative images of MG63 adhering atop hydrogels from agarose solutions cured at different temperatures; scale bar is 100 µm. b,c) Total cell number/hydrogel area and cell spread area on hydrogels from agarose cured at different temperatures. Data are reported as mean ± s.d., *n* = 25 images analyzed in (b) and *n* = 210 cells in (c). Statistics: **, *p* < 0.01; ***, *p* < 0.001; ****, *p* < 0.0001; ns, not significant (one‐way ANOVA followed by Dunnett's Multiple Comparison post hoc test in the plot (b) and by Tukey's Multiple Comparison post hoc test in the plot (c). d) Immunostaining of F‐actin and vinculin for MG63 cells on different agarose hydrogels; scale bars are 100 µm, zoom‐in/inset 10 µm.

Hydrogels of agarose cured at different temperatures were investigated as potential substrates enabling cell adhesion and spreading. We used a human osteosarcoma cell line, that is MG63, as a model.^[^ ^10^, ^26^ ^]^ When MG63 are plated on the hydrogels using a serum‐supplemented cell culture medium, they behave differently, with good adhesion to substrates developed from 121, 85, and 60 °C temperature‐cured agarose solutions (**Figure** **5**a). Among them, the number of cells/mm^2^ is significantly higher in the case of the 85 °C sample (Figure 5b). On the contrary, when the curing temperature is lowered to as low as 42 °C, a marked decrease in cell adhesion is observed. It is striking that there is a correlation between the curing temperature of agarose and the spreading of the cells, which decreases as the curing temperature lowers (Figure 5c). This is consistent with the immunostaining analyses of MG63, which show well‐stretched actin fibers in the case of the hydrogel cured at 121 °C, which gradually relaxes as the curing temperature is reduced (Figure 5d). Furthermore, we observe differences with respect to the formation of vinculin‐rich focal adhesions. Vinculin appears to be well assembled and organized in a belt‐like configuration in hydrogels formed from agarose cured at 121 °C as evidenced in the inset of Figure 5d. When the curing temperature is gradually lowered, vinculin appears less organized and shows more punctate structures. Overall, our results suggest that the curing temperature of agarose affects the ability of cells to adhere to the substrate and their spreading through vinculin‐rich adhesions with different geometry and size.

## 3 Discussion

**Figure 6:**
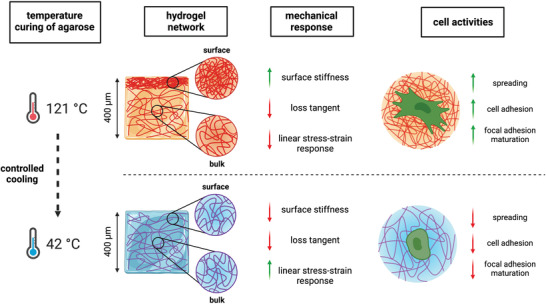
Controlled quenching of agarose defines hydrogels with tunable structural, bulk mechanical, surface nanomechanical, and cell response in 2D cultures. Schematic cartoon summarizing what was identified in this study. To reduce rapid cooling after heating, agarose solution is cured at different temperatures before hydrogel formation at room temperature. This has implications for i) the final hydrogel structure, with an accumulation of the biopolymer at the surface when the curing temperature is set high, and a more homogeneous network when the curing temperature is set low; in both cases, the surface is porous and hydrophilic; ii) the final mechanical response of the hydrogel bulk and surface, with adjustable stiffness and extension of the linear stress‐strain region, but not viscoelasticity (in terms of loss tangent or stress relaxation); iii) the response of the cells, with strong adhesion, spreading and assembly of the focal adhesions when the curing temperature is set high, and less adhesion, spreading and build‐up of the focal adhesions when the curing temperature is set low.

Taken together, our results show that the curing temperature of the agarose solution is crucial for the assembly of the final hydrogel network. This affects the nanomechanical properties of the surface and the mechanical response of the bulk. Furthermore, we have shown that cell adhesion, spreading, and focal adhesion maturation can be modulated by varying this experimental parameter (**Figure** **6**). Although this study does not aim to provide structural insights into the gelation mechanisms of agarose upon cooling, it is significant considering that quenching plays a crucial role in the formation of hydrogels, the kinetics and mechanism of which have been debated for years, especially due to the different experimental protocols used. While it is generally assumed that gelation occurs by liquid–liquid phase separation of agarose on cooling,^[^ ^27^, ^28^, ^29^ ^]^ the two main proposed mechanisms for hydrogel formation are spinodal decomposition or nucleation and growth. The controlled quenching rate has helped to decipher that self‐assembly of agarose proceeds through an induction phase in which gelation is initiated by a nucleation and growth mechanism leading to the formation of many nuclei consisting of a polymer‐rich phase dispersed in the solvent. The latter is followed by local coagulation of the polymer‐rich phases approaching the global minimum free energy of the whole system.^[^ ^7^ ^]^ In view of our results, it can therefore be expected that shifting the curing of agarose from 121 to 42 °C leads to a progressive control of quenching that influences, in turn, the self‐assembly of the biopolymer.

In this study, a combination of microscopy techniques has shown that agarose self‐assembles into inhomogeneous networks, with a large amount of polymer on the surface of the hydrogel, albeit at different depths depending on the curing temperature (Figure 1). This inhomogeneity can be drastically improved if the curing temperature is set to 42 °C, resulting in a more homogeneous distribution of agarose in the first 400 µm‐depth. Although this has no effect on the response of the hydrogels to a compressive load (Figure 2), it clearly affects the rheological performance at large deformations and the surface mechanics, as shown by nanomechanical experiments with AFM and the Piuma nanoindenter (Figures 2 and 3). Regarding surface properties, the lower the curing temperature of agarose solutions, the lower the stiffness. This can be explained by the different local concentrations of agarose reflecting the biopolymer distribution profiles shown in Figure 1c. The higher elastic response of the hydrogel surface in the case of curing at 121 °C is thus due to the higher network connectivity (crosslink density), given *E*∝*ρ*,^[^ ^30^ ^]^ and which decreases progressively as the curing temperature is reduced. Surface viscoelasticity in terms of loss tangent, on the other hand, shows no significant differences, which means that both the storage modulus and the loss modulus proportionally lower when decreasing the curing temperature (Figure 3b). While most hydrogels used to mimic the extracellular matrix for cell mechanobiology studies exhibit the same stiffness but different viscoelasticity,^[^ ^31^, ^32^, ^33^ ^]^ our hydrogels are peculiar in that they show the exact opposite behavior. Living tissues in the human body manifest different stiffness but are viscoelastic.^[^ ^34^, ^35^ ^]^ Therefore, our results could be very useful to provide 2D extracellular matrix substrates with controlled mechanics and to study the role of stiffness on cell response independent of viscoelasticity.

In this sense, we have studied the effects of this set of viscoelastic substrates on some key cell‐fate decisions. Although agarose can be functionalized with biomolecules by standard chemistry to promote cell‐substrate interactions,^[^ ^36^, ^37^ ^]^ we exploit serum proteins to form a coating on the hydrogel surface (Figure S6, Supporting Information) that enhances cell adhesion. When MG63 are plated on hydrogels from agarose cured at different temperatures, they show a different response in terms of the number of cells adhering to the substrate, spreading, and focal adhesion size and geometry (Figure 5). All these hydrogels have a surface rigidity well above the threshold for stiffness (≈5 kPa) that triggers the mechanotransduction cascade in purely elastic substrates.^[^ ^38^ ^]^ Although agarose hydrogels are viscoelastic, the contribution of viscous dissipation is very small (loss tangent < 10%). Therefore, the maturation of vinculin on agarose hydrogels is justified by a similar stiffness‐sensing as on polyacrylamide substrates. This is consistent with the observations on the increase of F‐actin stress fibers and cell spreading when the curing temperature is increased from 42 to 121 °C. The significant increase in cells attaching to the hydrogel when agarose is cured at 85 °C is tentatively explained by the very low roughness of these hydrogels, which could increase the local engagement of anchoring points on the relative microscale (Figure 4). On the other hand, the significant reduction in cells attaching to the hydrogel when agarose is cured at 42 °C can be interpreted in terms of reduced mechanical feedback due to lower agarose density at the surface (Figure 1),^[^ ^39^ ^]^ which may affect the bundling of integrins that is essential for promoting cell adhesion.^[^ ^10^, ^38^ ^]^ Overall, these results are very encouraging and lay the groundwork for further experiments to understand the molecular basis of such behavior.

In summary, our results reveal a material strategy for the development of viscoelastic agarose hydrogels with tunable structural, bulk mechanical, and surface nanomechanical properties suitable for the culture of cells in 2D and possibly also in 3D. Our results will have a major impact on the field of mechanobiology and pathology. In this context, recapitulating the stiffness and viscoelasticity of living or aberrant tissues in extracellular matrix mimics is a fundamental step toward achieving solid breakthroughs in this field. Furthermore, our results have helped to clarify conflicting findings on the use of agarose as a biomaterial for cell culturing. The effect of temperature curing of agarose solution has been largely overlooked and can now be used as an effective tool to study the interplay between cells and this set of biomaterials.

## 4 Experimental Section

### Agarose Source

Agarose was purchased from Euroclone, Italy (code EMR920500). The physical/chemical characteristics of the agarose are the following: total content of agaropectins (in terms of ashes) = 0.6% w/w; gelling temperature = 34 °C; rotational viscosity at 60 °C = 14 mPa s; total methylation = 7.4%.^[^ ^21^ ^]^

### Synthesis of Atto Rho101 NHS Ester‐Labeled Agarose

300 mg of agarose were dissolved in 15 mL of deionized water and the resulting mixture was stirred at ≈95 °C for 15 min to promote agarose solubilization. Then, the temperature was cooled down to 60 °C, and 125 µL of Atto Rho101 NHS ester (Sigma, USA) dissolved in DMSO at 2 mg mL^−1^ was added to the solution. The reaction mixture was stirred for 30 min and in dark conditions. The mixture was then poured into a Petri dish and let to gel at room temperature. Next, the labeled‐agarose hydrogel was cut into small pieces, extensively washed in deionized water, frozen overnight at −20 °C, and thawed the day after. Freeze‐thaw steps were repeated thrice to eliminate most of the solvent. The labeled agarose was finally vacuum‐dried at 60 °C and milled in a mortar. The labeled agarose (20% w/w) was used for the preparation of hydrogels intended for the analysis of biopolymer distribution from the surface to the inner part of the hydrogel by confocal microscopy.

### Temperature Curing of Agarose Solutions for Hydrogel Preparation

Agarose powder at a final concentration of 1% w/V was added to MilliQ deionized water. The resulting mixture was sealed in glass vials to prevent evaporation of the solvent and autoclaved at 121 °C for 15 min. Temperature curing of the agarose solutions was performed as follows: after autoclaving, part of the agarose solution was immediately placed in cylindrical supports and cooled at room temperature. This corresponded to the case of curing at 121 °C. The remaining agarose solution was incubated in a water bath pre‐warmed at 85 °C, using a probe to properly control the temperature. After reaching thermal equilibrium, the following sequence was followed: 10 min at 85 °C, then part of the solution was poured into cylindrical supports (85 °C case); lowering the temperature to 60 °C, 10 min at 60 °C, then part of the solution was poured into cylindrical supports (60 °C case); lowering the temperature to 42 °C, 10 min at 42 °C, then the remaining solution was poured into cylindrical supports (42 °C case). Finally, the hydrogels were incubated for 24 h at 37 °C to equilibrate the system^[^ ^7^ ^]^ under water‐saturated conditions to prevent evaporation of the solvent and then analyzed.

### Rheological Characterization

Rheological characterization of the hydrogels (20 mm in diameter, 2–2.5 mm thick) was performed by means of a controlled stress rheometer HAAKE MARS III operating at 37 °C using a shagreened plate‐plate apparatus (“HPP20 profiliert,” diameter 20 mm) as the measuring device. The experimental conditions for setting the measurements, frequency sweep, short and long stress sweep tests were performed as previously described.^[^ ^10^ ^]^

### Uniaxial Compression Characterization

Uniaxial compression of the hydrogels (16 mm in diameter, 17 mm thick) was performed by means of a universal testing machine (Mecmesin Multitest 2.5‐i) equipped with a 100 N load cell. A compression speed of 1 mm min^−1^ was applied to determine the initial elastic modulus in the strain range of 1–3%. Stress‐relaxation tests were conducted by applying a constant strain of 15% for 300 s. The time needed to relax the stress to half of the initial value, *τ*_0.5_, was considered as a parameter to compare hydrogels.^[^ ^23^ ^]^

### Atomic Force Microscopy

AFM measurements on the hydrogel surface were performed on an MFP‐3D Bio instrument (Oxford Instruments – Asylum Research). The topographic images were acquired in MilliQ water in contact mode using NSC‐36 cantilevers (radius of curvature < 10 nm, spring constant 0.6 < *k* < 2 N m^−1^). For the analysis of roughness, 20 × 20 µm^2^ images (from 3 to 5 per sample at different locations) were acquired at 256 × 256 pixels. AFM force‐distance curves were acquired in MilliQ water on the same instrument using pre‐calibrated cantilevers with beads from Novascan (diameter 2.5 µm, spring constant 0.06–0.07 N m^−1^). Force‐distance curves were acquired until a 5 nN force was detected with an indentation ranging from 100 to 500 nm depending on the different samples. 4 to 6, 4 × 4 force maps, were acquired in different locations for a total of 64 to 96 curves analyzed per sample. Analysis of force‐distance curves was performed using AtomicJ after calibration of the sensitivity of the instruments by performing force‐distance curves on a glass slide in liquid. The Young's Modulus was extracted using a classic Hertzian model using as fixed parameters the radius of curvature of the sphere and the Poisson's ratio (*P* = 0.5).

### Piuma Nanoindenter

Indentation experiments of hydrogels surface were also performed using the Piuma nanoindenter (Optics11 Life, Amsterdam, The Netherlands) and a cantilever‐based probe with a spherical tip radius of 26 µm and a cantilever stiffness of 0.23 N m^−1^. Measurements were conducted using the controlled indentation mode, at an indentation depth of 5 µm. The samples were measured with a 3 × 3 matrix scan with a 50 µm distance, resulting in nine measurement points per hydrogel. From the obtained load‐indentation curves, the Hertzian contact model was used to calculate the effective Young's modulus. DMA measurements were performed to obtain storage and loss moduli, using a frequency of 1 Hz and an oscillation amplitude of 100 nm. All measurements were performed at room temperature and in distilled water.

### Environmental Scanning Electron Microscopy

Hydrogels were mounted on aluminum stubs covered with double‐sided carbon tape and analyzed with a scanning electron microscope (Quanta250 SEM, FEI, Salem, OR, USA) working in environmental conditions, using an acceleration voltage of 30 kV and a working distance of 10 mm.

### Contact Angle Measurements

Contact angle measurements were performed by means of an optical stereomicroscope (MZ16, Leica Microsystems GmbH) equipped with a digital camera (DFC320, Leica Microsystems GmbH) and a 45° tilted mirror. A droplet of deionized water (4 µL) was placed atop the hydrogel surface and let to rest for 1 min. The profile of droplets was next recorded. Image‐Pro Plus 6.2 software (Media Cybernetics, Inc.) was used to acquire, process the images, and measure the contact angle.

### Cell Culture

Human osteosarcoma MG63 (ATCC CRL‐1427) was cultured in Dulbecco's modified Eagle's Medium high glucose with 0.584 g L^−1^ l‐glutamine and 0.11 g L^−1^ sodium pyruvate (EuroClone, Italy), supplemented with 10% heat‐inactivated fetal bovine serum (Cat. n° ECS5000L; Lot. n° EUS00K1, Euroclone, Italy) and 1% penicillin/streptomycin (EuroClone, Italy), in a humidified atmosphere of 5% CO_2_ at 37 °C.

### Plating of Cells Atop Hydrogels

For this set of experiments, agarose powder was dispersed in PBS prior to autoclaving. After temperature curing of agarose, 400 µL of the solutions were distributed into 24‐well plates and allowed to rest for 30 min at room temperature to promote gelation. Finally, the hydrogels were incubated overnight at 37 °C in water‐saturated conditions. Cells were plated at a density of 15 000 cells/cm^2^ using 1.6 mL of complete cell culture medium/well, giving a final *V*_PBS_/*V*_medium_ ratio of 20:80 V/V. The cells were incubated overnight in a humidified atmosphere with 5% CO_2_ at 37 °C.

### Assessment of Cell Adhesion and Spreading

After overnight incubation, the cell culture medium was discarded and hydrogels were washed extensively with PBS in order to remove non‐adherent cells. Then, 300 µL/well of PBS was added and the number of adherent cells/mm^2^ and cell spreading was quantified by Fiji‐ImageJ software (multi‐point and freehand selection tools, respectively) from images acquired through a Nikon Ti Eclipse inverted bright‐field microscope equipped with an Intensilight Epi‐fluorescence Illuminator, a Perfect Focus 3 system, and a Plan Fluor 10x DIC L N1 objective. The acquisition was performed with a DS‐Qi2 16 Mpixel camera (Nikon).

### Cell Immunostaining and Image Analysis

After overnight incubation, the cell culture medium was discarded and hydrogels were washed extensively with PBS in order to remove non‐adherent cells. Then, hydrogels were punched into small disks (9 mm in diameter, 2.5 mm thick) and moved in a clean 48‐well plate. Immunostaining was performed as previously described,^[^ ^10^ ^]^ using the following primary antibody: vinculin antibody (dilution 1:200 or 5 µg mL^−1^, V9264, Sigma); secondary antibody: mouse IgGk light chain (dilution 1:250 or 1.6 µg mL^−1^, sc‐516179, Santa Cruz). For the visualization of F‐actin filaments and nuclei, cells were counterstained with Phalloidin Fluorescein Isothiocyanate Labeled (P5282, Sigma, 1 µg mL^−1^ in PBS) and Hoechst (33 258, Invitrogen, 5 µg mL^−1^ in PBS), respectively. Images from immunofluorescence and cellular staining experiments were acquired using a Nikon C1si confocal microscope (Nikon, Tokyo, Japan), containing 488 (argon), 408, and 561 nm (diode) lasers. The light was delivered to the sample with an 80/20 reflector. The system was operated with a pinhole size of one airy disk. Electronic zoom was kept at minimum values for measurements to reduce potential bleaching. For the different fields collected, 20× Plan Apo objective was used, saving a series of optical images with 2.2 µm z‐resolution step size. Images in various conditions were captured under identical acquisition settings in order to allow comparison of fluorescent intensity and were processed for maximum z‐projection by using Fiji‐ImageJ (NIH, Bethesda, USA).

### Synthesis of FITC‐Labeled BSA

400 mg of bovine serum albumin (BSA, Sigma) were dissolved in 10 mL of NaHCO_3_ 30 mm. Then, 500 µL of a fluorescein‐isothiocyanate (FITC, Sigma) solution (2 mg mL^−1^ in pure ethanol) was added to the BSA solution. The reaction mixture was stirred for 24 h at room temperature and under dark conditions. The mixture was then dialyzed at 4 °C against NaHCO_3_ 30 mm (two shifts, *V* = 500 mL) and deionized water (three shifts, *V* = 1 L). All steps were carried out under dark conditions. Finally, the solution was freeze‐dried.

### Evaluation of BSA Absorption on the Hydrogel Surface

Hydrogels of agarose cured at different temperatures were prepared in PBS buffer as described above. They were then incubated O/N at 37 °C with 1.6 mL of FITC‐labeled BSA (0.5 w/V dispersed in PBS). The next day, the BSA solution was discarded and the hydrogels were washed extensively with PBS to remove non‐adherent BSA and punched to obtain 6 mm diameter discs. They were then placed on a coverslip and analyzed by confocal microscopy using a Plan Fluor 20× objective. A series of images were saved at 266 × 266 µm with a 4 µm z‐resolution step size. The images were acquired with identical acquisition settings to allow comparison of fluorescence intensities between the analyzed samples and were processed with Fiji‐ImageJ. Fluorescence intensity was quantified within 8 µm depth from the hydrogel surface by measuring the intensity of 8–10 different 10 µm × 10 µm fields using the ImageJ ROI manager tool.

### Statistical Analysis and Software

Statistical comparisons and graphical elaborations were carried out using GraphPad Prism software. A one‐way analysis of variance (ANOVA) was performed, followed by a Tukey's or Dunnett's post hoc test to assess differences between the different groups. Differences were considered significant if the *p*‐value was less than 0.05. The cartoons were created with BioRender.com.

## Conflict of Interest

The authors declare no conflict of interest.

## Author Contributions

Conceptualization: P.S.; Methodology: F.P., P.P., J.P., E.M., L.B., D.P., G.B., I.D., and P.S.; Software: F.P., G.B.; Investigation: F.P., P.P., J.P., L.B., D.P., and G.B.; Data curation: F.P., P.P., L.B., D.P., and P.S.; Supervision: I.D., P.S.; Writing the manuscript: P.S.

## Supporting information

Supporting Information

## Data Availability

The data that support the findings of this study are available from the corresponding author upon reasonable request.
