# Supplementary material for: Controlled Quenching of Agarose Defines Hydrogels with Tunable Structural, Bulk Mechanical, Surface Nanomechanical, and Cell Response in 2D Cultures
Source: Adv Healthc Mater. 2023 Jul 9;12(26):2300973. doi: 10.1002/adhm.202300973 (PMC11468619; doi:10.1002/adhm.202300973)
Supplement: Supplementary file 1 — Supporting Information [file ADHM-12-2300973-s001.pdf]

ADVANCED HEALTHCARE MATERIALS

Supporting Information

for *Adv. Healthcare Mater.*, DOI 10.1002/adhm.202300973

Controlled Quenching of Agarose Defines Hydrogels with Tunable Structural, Bulk Mechanical, Surface Nanomechanical, and Cell Response in 2D Cultures

*Francesco Piazza, Pietro Parisse, Julia Passerino, Eleonora Marsich, Luca Bersanini, Davide Porrelli, Gabriele Baj, Ivan Donati and Pasquale Sacco**

Supporting Information

Controlled quenching of agarose defines hydrogels with tunable structural, bulk mechanical, surface nanomechanical and cell response in 2D cultures

Francesco Piazza, Pietro Parisse, Julia Passerino, Eleonora Marsich, Luca Bersanini, Davide Porrelli, Gabriele Baj, Ivan Donati, and Pasquale Sacco*

Mr. F. Piazza, Ms. J. Passerino, Prof. G. Baj, Prof. I. Donati, Prof. P. Sacco
Department of Life Sciences
University of Trieste
Via Licio Giorgieri 5, I-34127 Trieste, Italy
E-mail: psacco@units.it

Dr. P. Parisse
NanoInnovation Lab, Elettra-Sincrotrone Trieste S.C.p.A., I-34149 Trieste, Italy
Istituto Officina dei Materiali (IOM-CNR), Area Science Park, I-34149 Trieste, Italy

Prof. E. Marsich
Department of Medicine, Surgery and Health Sciences
University of Trieste
Piazza dell'Ospitale 1, I-34129 Trieste, Italy

Dr. L. Bersanini
Optics11 Life
Hettenheuvelweg 37-39, 1101 BM, Amsterdam, The Netherlands

Dr. D. Porrelli
Interdepartmental Centre for Advanced Microscopy, Department of Life Sciences
University of Trieste
Via Alexander Fleming 31/A, I-34127 Trieste, Italy

Additional Figures.

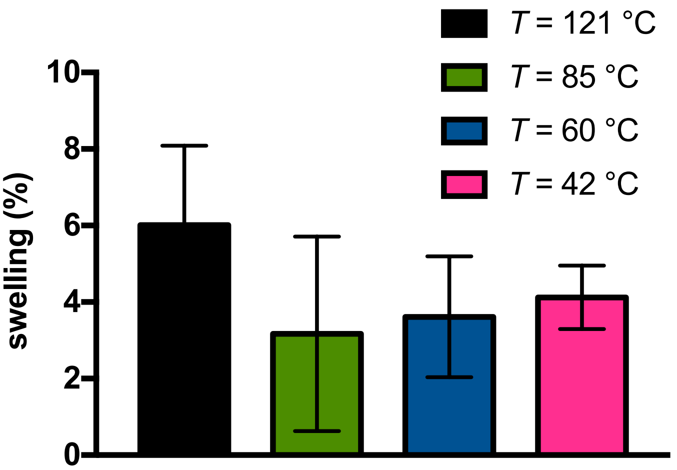

Figure S1. Swelling behavior in MilliQ deionized water of agarose hydrogels developed at different curing temperature. Data are reported as mean ± s.d., n = 5 hydrogels analyzed for each experimental condition. Experimental conditions: [agarose] = 1% w/V, MilliQ deionized water as solvent, incubation time = 3 days.

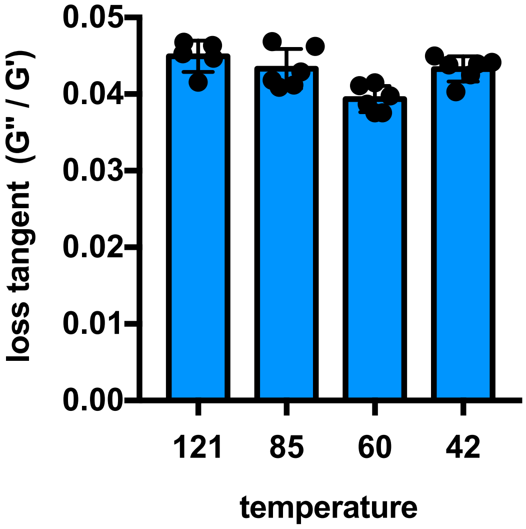

Figure S2. Loss tangent recorded at 1 Hz for agarose hydrogels developed at different curing temperature. Data are reported as mean ± s.d., n = 5 - 6 hydrogels analyzed for each experimental condition. Experimental conditions: [agarose] = 1% w/V, MilliQ deionized water as solvent.

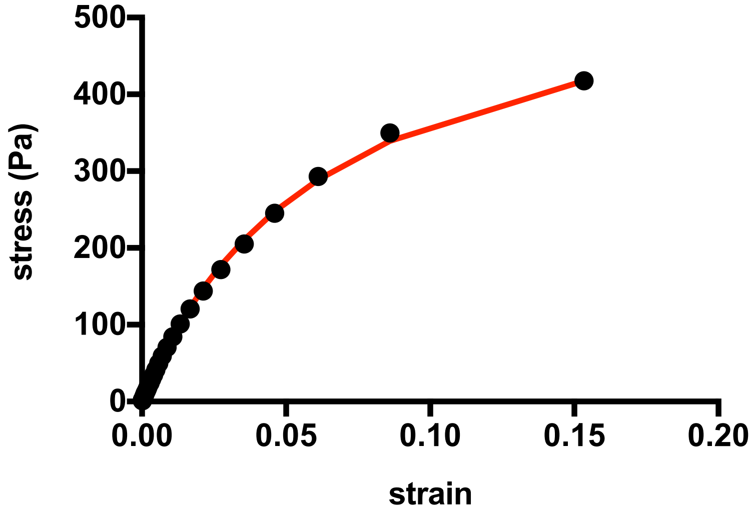

Figure S3. Typical softening behavior under oscillatory mechanical stimulation of agarose hydrogels developed at different curing temperatures. The black dots represent the experimental points, while the red solid line is the best fit obtained by equation 4 in the main manuscript. Experimental conditions: [agarose] = 1% w/V, MilliQ deionized water as solvent.

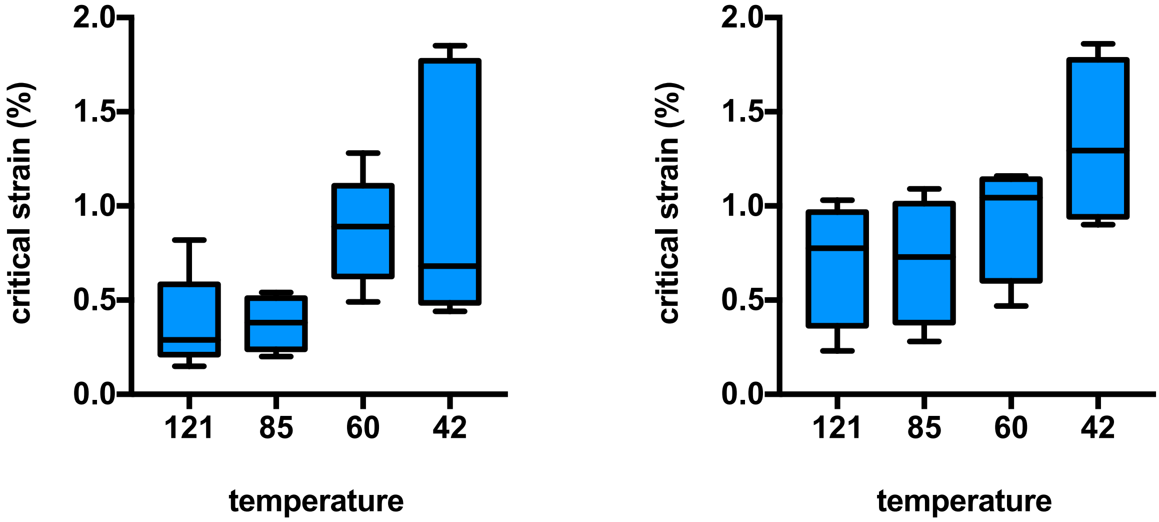

Figure S4. Critical strain where the linear stress-strain response of the hydrogels obtained from agarose cured at different temperature ends. Experimental conditions: [agarose] = 1% w/V, PBS buffer (left) and MilliQ deionized water (right) as solvent. Physical-chemical properties of agarose used in the plot at left: T_{gel} = 34 °C; rotational viscosity at 60 °C = 14 mPa s; total methylation = 7.4%; agaropectin content = 0.6% w/w; right: T_{gel} = 40 °C; rotational viscosity at 60 °C = 24 mPa s; total methylation = 12.8%; agaropectin content = 1.1% w/w. Data are reported as box and whiskers (min to max), n = 4 - 9 hydrogels analyzed for each experimental condition.

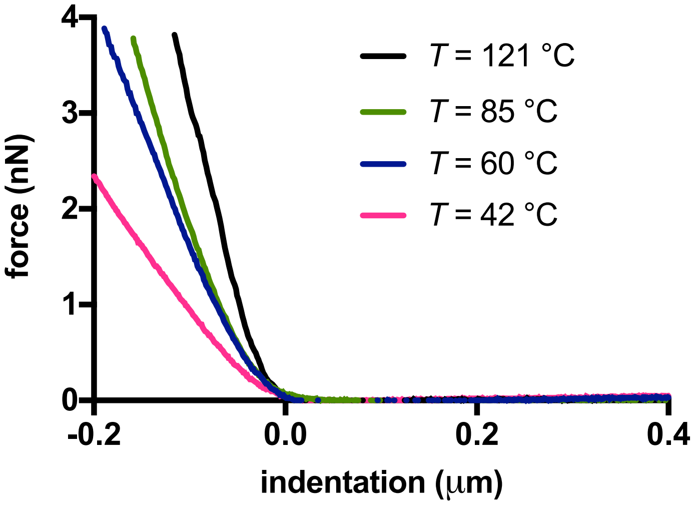

Figure S5. Sample-case indentation curves obtained through atomic force microscopy of agarose hydrogels developed at different curing temperatures. Experimental conditions: [agarose] = 1% w/V, MilliQ deionized water as solvent.

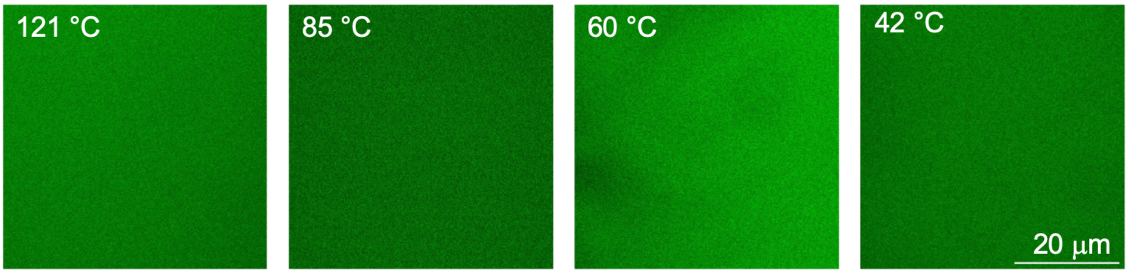

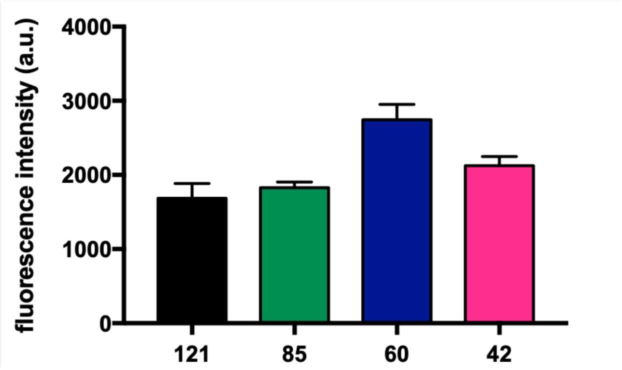

Figure S6. Evaluation of the absorption of bovine serum albumin (BSA), the major component of serum proteins (*Biotechnol. Prog.* 2006, 22, 1294−1300; *Journal of Biological Methods*, 2016, 3(4), e51), on the hydrogels surface from agarose cured at different temperature. 50 x 50 μm fields from confocal microscopy analysis (top) and fluorescence intensity (bottom) quantified within 8 μm depth from the hydrogel surface by measuring the intensity of 8 - 10 different 10 μm × 10 μm fields using the ImageJ ROI manager tool; data are reported as mean ± s.d.
